# A Resource-Efficient CNN-Based Method for Moving Vehicle Detection

**DOI:** 10.3390/s22031193

**Published:** 2022-02-04

**Authors:** Zakaria Charouh, Amal Ezzouhri, Mounir Ghogho, Zouhair Guennoun

**Affiliations:** 1 ERSC Team, Mohammadia Engineering School, Mohammed V University, Rabat 10 090, Morocco; amal.ezzouhri@uir.ac.ma (A.E.); zouhair@emi.ac.ma (Z.G.); 2 TICLab, College of Engineering and Architecture, International University of Rabat, Rabat 11 100, Morocco; mounir.ghogho@uir.ac.ma; 3 School of Electronic and Electrical Engineering, Faculty of Engineering, University of Leeds, Leeds LS2 9JT, UK

**Keywords:** convolutional neural network, background subtraction, monitoring, object detection, vehicle detection

## Abstract

There has been significant interest in using Convolutional Neural Networks (CNN) based methods for Automated Vehicular Surveillance (AVS) systems. Although these methods provide high accuracy, they are computationally expensive. On the other hand, Background Subtraction (BS)-based approaches are lightweight but provide insufficient information for tasks such as monitoring driving behavior and detecting traffic rules violations. In this paper, we propose a framework to reduce the complexity of CNN-based AVS methods, where a BS-based module is introduced as a preprocessing step to optimize the number of convolution operations executed by the CNN module. The BS-based module generates image-candidates containing only moving objects. A CNN-based detector with the appropriate number of convolutions is then applied to each image-candidate to handle the overlapping problem and improve detection performance. Four state-of-the-art CNN-based detection architectures were benchmarked as base models of the detection cores to evaluate the proposed framework. The experiments were conducted using a large-scale dataset. The computational complexity reduction of the proposed framework increases with the complexity of the considered CNN model’s architecture (e.g., 30.6% for YOLOv5s with 7.3M parameters; 52.2% for YOLOv5x with 87.7M parameters), without undermining accuracy.

## 1. Introduction

One of the fundamental pillars of road safety strategies is deploying Automated Vehicular Surveillance (AVS) systems to monitor driving behaviors and detect dangerous driving patterns. Fast and accurate detection and classification of vehicles are necessary for an effective AVS system. Cameras and machine vision are crucial in AVS systems as videos contain rich information about the road and vehicles. However, sophisticated video analytics methods based on Deep Learning (DL) are generally computationally demanding, which may hinder their real-time implementation. This motivates the development of techniques to reduce their complexity.

Vehicle detection methods used by current AVS systems rely mostly on Background Subtraction (BS) methods because of their low complexity. BS is a motion-based method that assigns each pixel in a video frame to either the background class (i.e., static objects) or the foreground class (i.e., moving objects). This method may produce poor detection results due to several reasons, such as: (1) object overlapping: depending on the of view, the projected image from the real domain may contain overlapped vehicles—multiple vehicles may thus be misclassified as one heavy vehicle; (2) parameter tuning: the performance of BS methods greatly depends on the threshold values set for their parameters; inadequate tuning lead to false detections or undetected vehicles; (3) dynamic background conditions: even when the camera is static, the background may change due to sudden illumination changes where intensities of pixels belonging to the background can significantly vary over very short periods thus leading to the misclassification of background pixels as foreground pixels [1]. In [2], we have proposed a machine learning-based solution to mitigate this problem. On the other hand, DL-based approaches are more reliable for detection and classification thus providing a good understanding of the scene. However, applying a CNN model to every video stream frame is computationally expensive and requires high-end computing units to be deployed. This paper proposes a framework combining BS and CNN-based detectors to minimize the number of convolutions needed to process a given frame, thus reducing the computational complexity without undermining the detection performance. The remainder of this paper is organized as follows. Section 2 discusses the related work. Section 3 presents the problem formulation. Section 4 presents the proposed framework, including the main component of the proposed framework and the overall architecture. Section 5 presents the experimental results. Finally, conclusions are drawn in Section 6.

## 2. Related Work

Several methods combining BS and DL for object detection have been proposed. In general, this combination can be divided into three categories: (i) using DL for background generation or enhancement and then performing the subtraction task using the generated background model, (ii) using DL for the subtraction process to generate the foreground, or perform the conventional process and use the generated foreground as input to a DL module that outputs an enhanced foreground, (iii) performing the background subtraction process conventionally and using DL to classify the detected moving objects.

### 2.1. DL for Foreground Generation

In [3], the authors proposed an algorithm that uses a sequence of ten consecutive frame-difference images as input to the CNN model. The latter is used to enhance the obtained results and then outputs the detected foreground objects. In [4], the authors proposed a foreground enhancement framework using a CNN-based semantic segmentation algorithm. The framework takes the mask generated by the BS algorithm as input and then produces a refined binary mask. In [5], the authors proposed a hybrid motion-based object detection method for detecting and tracking small and fast-moving drones. Information on the motion pattern from the motion-based method and the object appearance from the appearance-based method are then combined. In [6], an encoder–decoder tool was proposed to deal with the foreground segmentation task. The proposed solution relies on an improved version of the VGG-16. The authors in [7] proposed a conditional Generative Adversarial Network (GAN)-based BS method. The network was designed to output a foreground mask by the GAN generator. In [8], the authors generated a background model using SuBSENSE and flux tensor algorithms, and then used a CNN model followed by a post-processing step to generate the foreground mask. The authors of [9] proposed a background subtraction approach based on CNN to process data provided by depth sensors. Firstly, background generation is done by the average background modeling method using depth values. The obtained result is then merged with the original image in a two-channel patch and fed to a CNN-based model. The final result is the foreground probability of each pixel.

### 2.2. DL for Background Generation

In [10], the authors proposed a deep probabilistic background-modeling framework using an unsupervised deep learning-based autoencoder architecture. The framework learns the background representation by looking for redundant regions from the video sequence to generate the background image. In [11], the authors proposed a conditional generative adversarial network to learn the nonlinear mapping between the input video frame and the background. The proposed method was shown to provide good performance on the background generation task. In [12], an end-to-end framework based on Generative Adversarial Network (GAN) has been proposed to generate dynamic background information in an unsupervised manner. Further, the simple pixel-level difference between the estimated background and the current video frame is calculated for the final segmentation of the foreground. In [2], the authors proposed a machine learning-based solution to automatically adjust the hyper-parameters of the background subtraction method, thus leading to an enhanced background detection model. In [13], the authors proposed a BS method using a CNN-based network to deal with the dynamic background problem. A simple background image generation was used with a CNN network, trained on scene-specific data, to classify the image pixels into either background or foreground. In [14], the authors proposed a deep auto encoder-based method. They used a cascade architecture of two deep autoencoder networks to model the background.

### 2.3. DL for Moving Object Classification

**Figure 1:**
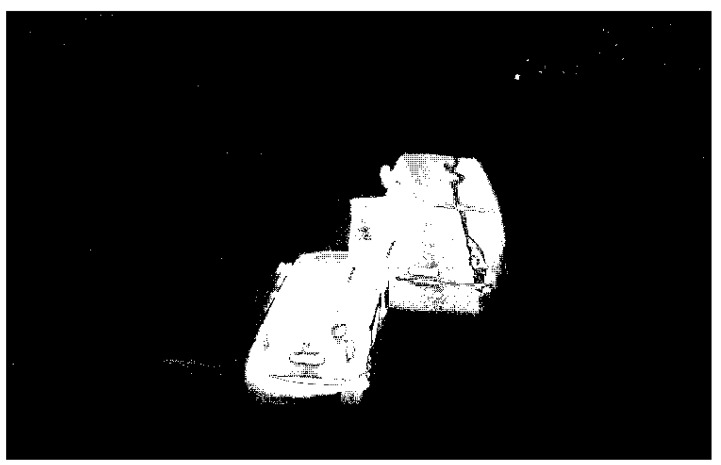
Illustration of a binary mask, obtained by GMM, which contains overlapped vehicles.

In [15], the authors proposed hybrid algorithms for object recognition, where Haar Cascade, Local Binary Pattern (LBP), and Histogram Oriented Gradients (HOG) algorithms were used for object detection, Convolutional Neural Network (CNN), and Artificial Neural Network (ANN) algorithms were used for classification. As a result, six hybrid structures were developed: Haar Cascade + CNN, LBP + CNN, HOG + CNN, Haar Cascade + ANN, LBP + ANN, and HOG + ANN. Experimental results showed that the accuracy of the Haar Cascade + CNN algorithm outperformed CNN and other hybrid algorithms. In contrast, LBP + CNN was the fastest. In [16], the authors combined BS and CNN to perform anomaly detection for pumping unit monitoring; the SuBSENSE BS method was adopted to extract the foreground objects in the video, and GoogLeNet classifier was used to identify the moving objects. In [17], the authors coupled a dynamic background modeling with DL classification to develop a scheme for human-animal detection from camera-trap images. First, they introduced a new block-wise background model, named Minimum Feature Difference (MFD), to model the background variation of the camera-trap sequences and generate the foreground object proposals. They then developed a region proposal verification to reduce the number of false alarms. Finally, they performed a complexity and accuracy analysis to construct a fast DL classification scheme to classify these region proposals into three categories: humans, animals, and background patches. The authors in [18] proposed a hybrid framework combining BS and CNN-based classification for person detection. This method, however, cannot be applied for precise object detection when considering the object overlapping case, that is, when the sub-image (image-candidate) proposed by the BS consists of a group of overlapped vehicles; see Figure 1. Indeed, the classifier looks for a unique object to be classified; so it fails to recognize all vehicles in the proposed image-candidate.

In order to study driving behavior by measuring behavioral metrics (e.g., speed, headway) using low-resource roadside devices, we aim to develop less complex but still accurate methods for detecting and classifying vehicles. The methods mentioned above focused on the subtraction’s pixel-wise binary classification ignoring either the execution time criterion, the overlapping problem mentioned previously, or both. This paper proposes a CNN-based object detection framework that significantly reduces the number of convolutions without sacrificing performance.

## 3. Problem Formulation

For AVS applications, stationary cameras are deployed by the roadside to monitor the passing vehicles. Recorded images often contain static and dynamic areas that are unnecessary for scene understanding algorithms; unnecessary static areas may include trees, buildings, and other static objects in the scene, and unnecessary dynamic areas are those which do not contain vehicles. We define the complexity of the CNN-based detection algorithm, denoted by *C*, as:

(1) $$C\left(I\right)=C_{cnn}\left(I\right)+C_{pp}\left(I\right)$$

where *I* is an image of a given shape, $C_{cnn}$ denotes the CNN complexity, and $C_{pp}$ denotes the complexity of the post-processing required by the detection algorithm to select suitable bounding boxes and classes from the model’s predictions (e.g., Non-maximum Suppression) representing the detected objects.

**Figure 2:**
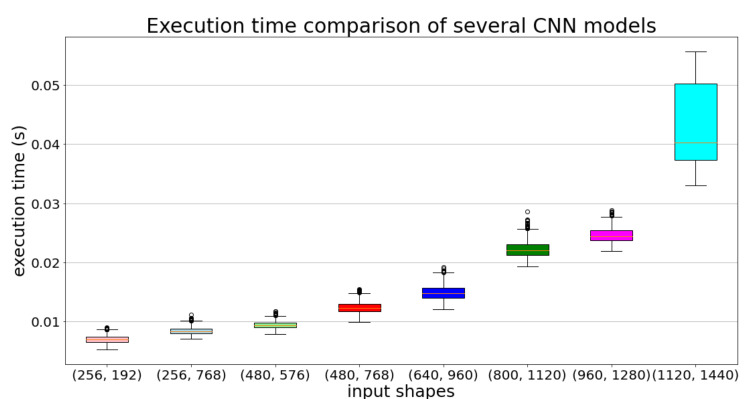
Inference time comparison for several CNN models with different architectures but sharing the same set of filters.

To illustrate the relationship between the image shape and the computational complexity, we collected a set of 900 images and defined eight several input shapes: 256 × 192, 256 × 768, 480 × 576, 480 × 768, 640 × 960, 800 × 1120, 960 × 1280, 1120 × 1440; then for each shape, we built the corresponding CNN models (based on the Tiny-YOLOv3 [19]) and post-processing modules. Figure 2 shows that the computational complexity of the detection task is correlated with the model’s input shape, as anticipated.

Motivated by the above observation, we infer that if we can specify the parts of the image to which the CNN should perform the convolution, we can reduce the computation complexity and the execution time, as described next.

Let $\left\{F_{i}\right\}$ and $\left\{B_{j}\right\}$ denote respectively a set of *n* relevant sub-images and a set of *m* irrelevant sub-images whose union forms the image *I*, i.e.,

(2) $$I=\left(\cup_{i=1}^{n}F_{i}\right)\;⋃\;\left(\cup_{j=1}^{m}B_{j}\right)$$

The overall complexity for the conventional CNN-based detection method can then be expressed as

(3) $$C\left(I\right)=\sum_{i=1}^{n}C_{\mathrm{cnn}}\left(F_{i}\right)+\sum_{j=1}^{m}C_{\mathrm{cnn}}\left(B_{j}\right)+C_{\mathrm{pp}}\left(I\right)$$

since the complexity of the convolution operator is proportional to the size of the input sub-image.

The idea behind our proposed method is first to determine relevant sub-images and then perform the detection task on these sub-images only with a view to reducing the overall complexity of the detection method.

Let g(I) denote the complexity of selecting relevant sub-images from the image *I*. The complexity of the proposed detection framework, denoted by $\overset{˘}{C}$, can thus be expressed as:

(4) $$\overset{˘}{C}\left(I\right)=\sum_{i=1}^{m}C_{cnn}\left(F_{i}\right)+g\left(I\right)+\sum_{i=1}^{n}C_{pp}\left(F_{i}\right)$$

The aim is to choose a low computational complexity algorithm for relevant sub-images detection so that $\overset{˘}{C}\left(I\right)$ is (much) lower than C(I) while keeping the same performance as the original CNN-based detection method. The complexity g(I) must thus satisfy the following constraint

(5) $$g\left(I\right)<\sum_{j=1}^{m}C_{cnn}\left(B_{j}\right)+C_{pp}\left(I\right)-\sum_{i=1}^{m}C_{pp}\left(F_{i}\right).$$

The reduction in computational complexity is measured by the difference between the right-hand side term and the left-hand term of the above inequality. In this paper, we propose to use the low-complexity BS approach to determine relevant sub-images.

## 4. The Proposed Framework

**Figure 3:**
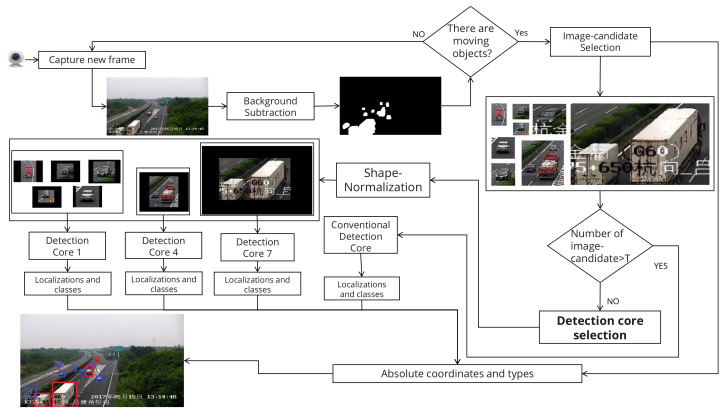
An example illustrating the application of the proposed framework to a video frame. The Background subtraction module generates eight image-candidates; in this example, the number of image-candidates is less than T (T = 10 in this study). Detection-cores 1, 4, and 7 are involved in this example.

The goal of our framework is to detect vehicles accurately while optimizing the execution time. This is done by first taking advantage of a low-complexity BS method to process the whole frame and eliminating non-interesting regions (i.e., regions without vehicles), then using a CNN-based detection-core on regions of interest for accurate vehicle detection. Next, we first describe the preprocessing phase consisting of BS and data preparation; then, we present the CNN-based detection-core and the main implementation details. Figure 3 illustrates the proposed framework through an example.

### 4.1. Background Subtraction

#### 4.1.1. Background

The acquired video sequence from a static surveillance camera contains static (i.e., the background) and moving (i.e., the foreground) objects. BS is a motion-based object detection method; it compares a given frame to the background to extract regions representing moving objects, i.e., vehicles in the scene. This study uses the Gaussian Mixture Model (GMM) as a BS method, which is a pixel-wise classification. The background is constructed over every pixel by generating Gaussian mixture models representing background intensities from a history window. The pixel assignment is done using a difference threshold—the pixel is compared to the background model, pixels exceeding the threshold parameter are classified as foreground pixels; the rest are classified as background pixels. While it is a frame-by-frame process, an update is then applied to the background model to consider the background modifications. The output is a binary mask of moving objects pixels’ locations in the original frame.

#### 4.1.2. Data Preparation

**Figure 4:**
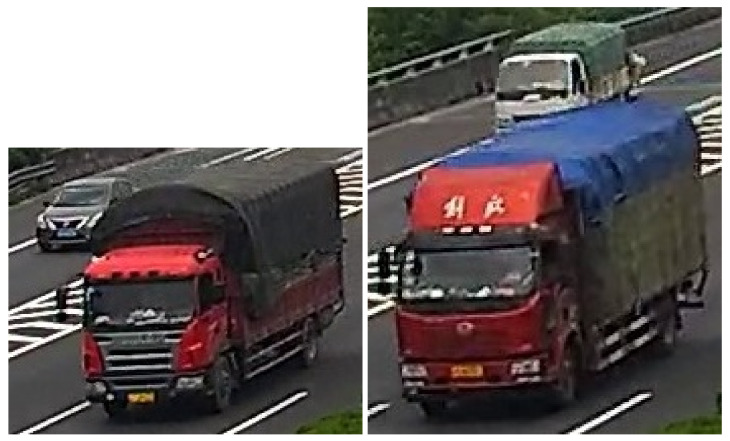
Illustration of image-candidates containing overlapping vehicles.

In addition to the GMM, the binary mask is conventionally processed to select all groups of white pixels (blob’ masks) to be classified using prior-specified shapes to valid moving objects or not. However, using a fully motion-based method may lead to consider non-vehicles related blob’s mask (e.g., humans, animals, a dynamic part from the background) as a vehicle. We then evaluate this blind method as non-sufficient for object detection, and we assume that an appearance-based method should be applied. We select for every blob’s mask its related image-candidate from the original frame to be fed to a preprocessing module for the CNN-based core. The image-candidates are of several shapes, and CNN models require a defined input shape; a simple method is to resize all image-candidates to a given shape to be processed by the CNN-based core, but as illustrated in Figure 4, one image-candidate may contain small and heavy vehicles. Resizing the image-candidate to a smaller shape may destroy the relevant features of the small car that uses the CNN-based core to recognize it, and resizing the image-candidate to a larger shape ends by adding more complexity to the process. We decided then to use multiple shapes, that is, multiple CNN-based cores.

As the BS-based module is used to reduce the number of convolutions, some frames are empty from vehicles. Then no image-candidate is selected, which permits the decision of even running the CNN-based core or not. We do not run the CNN-based core on empty frames, which reduces the whole complexity when eliminating some frames (see comparison result in Section 5).

### 4.2. CNN-Based Core

#### 4.2.1. Background

Fully DL-based solutions use appearance to recognize objects in the image instead of detecting moving objects. Thus, in contrast to the fully motion-based methods, the appearance-based methods can detect and distinguish two overlapped vehicles.

In [20], the authors proposed an advanced region-based CNN detector—the Faster R-CNN detector; its robustness was due to the introduction of the Region Proposal Network (RPN) to propose to the classifier relevant regions. The RPN uses the anchor mechanism, which is a set of handcrafted boxes of several sizes. The main contribution was merging the RPN and the Fast RCNN into one network by sharing the feature extraction block of convolution layers. In [21], the authors improved the Faster R-CNN and proposed the Region-based Fully Convolutional Network (RFCN); the improvement was due to the introduction of the Positive-Sensitive Score Maps. Another family of object detectors is the single-shot family of algorithms, such as EfficientDet [22] and the YOLO family [19,23,24,25]. To the best of our knowledge, YOLOv5 represents the state of the art when it comes to detection algorithms and offers the best combination of execution speed and detection accuracy as the architectures progress from YOLOv5s (a lite model) to YOLOv5x (a deep model). In this study, we used the four YOLOv5 architectures as the base models of the detection cores.

Appearance-based classifiers use filters to extract relevant features from an image; the activated set of filters refer to a prior-defined class. CNN classifiers are appearance-based and use mostly multiple convolution layers to classify the image. Every convolution layer comprises a set of filters of several shapes (e.g., 1 × 1, 3 × 3, 5 × 5) trained to be activated on a given feature (e.g., tires, doors, and lamps for a vehicle) to generate the so-called feature-map. The feature map is fed to an artificial neural network (ANN) using fully connected and activation layers to be classified. CNN-based detection algorithms are used to classify multiple objects and detect their locations in an image. The appearance property is involved instead of using the motion property over a history window. CNN detectors use anchors, which are a set of predefined shapes of windows to extract regions from the image, to be classified using the CNN-based classification technique, and then getting objects’ locations and classes. The proposed framework aims to reduce the complexity of the object detection process while using CNN. Instead of analyzing anchors containing empty areas (i.e., background), we propose to run the detection-core directly on regions of interest representing relevant image-candidates. As mentioned before, we need to define multiple detection-cores, which means that, for each image-candidate’s shape, we use the appropriate detection-core. The detection-core takes as input the image-candidate and outputs all detected vehicles’ coordinates and types. Subsequently, we apply a transformation to obtain the absolute coordinates (i.e., the coordinates in the original image).

#### 4.2.2. Preprocessing

The image-candidates proposed by the BS step refer to the coordinates of the moving object in the image. Before running the CNN-based detection-core on an image-candidate, we should prepare the image-candidate to allow the detection-core to recognize the object representation and features, mainly its boundaries. We then add some pixels from the original image that surround the image-candidate.

**Figure 5:**
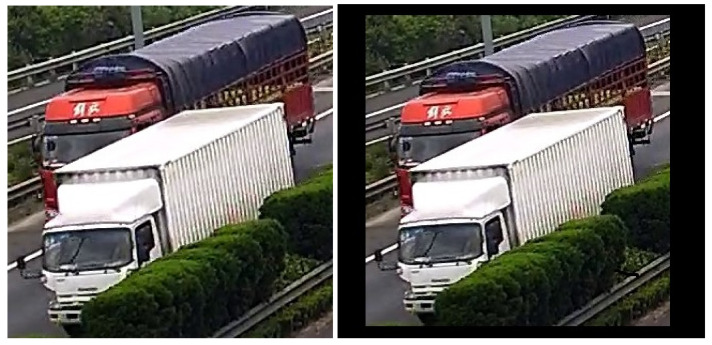
The shape-normalization consists of adding black pixels to an image (**left**) to obtain a new image (**right**) with the desired shape.

While CNN requires a defined input shape, we defined representative input shapes (the mechanism is explained in Section 4.2.3); all the received image-candidates from the previous step are then shape-normalized. Thus, we use the padding technique, which consists of adding zero-intensities pixels to every image-candidate so it can reach the desired shape (see Figure 5).

#### 4.2.3. Model Design

Detection-cores use convolution and pooling layers to extract features from images. The convolution and pooling output shapes are obtained based on the parameters of the layers (i.e., stride, padding, and filter size). Let the output shape be *O* × *O*, the input shape be *I* × *I*, the kernel size be *K* × *K*, the padding be *P* × *P*, and the stride be *S* × *S*,

(6) $$O=\frac{I-K+2P}{S}+1$$

where *O*, *I*, *K*, *P* and *I* are integers, thus, reducing the image’ input shape affects the whole architecture. Table 1 illustrates a comparison of the first eight convolution layers of two YOLOv5s-based detection-cores architectures with different input shapes.

The vehicles in the original image and the image-candidate have equal dimensions. When applied to the two images (i.e., the original and the image-candidate), a CNN-based filter gives the same result, thanks to the principle of convolution. Thus, the filters obtained for a detection-core (in particular the detection-core with largest input shape) can be reused for the other detection-cores because the model training process aims to adjust the filters used in each layer, regardless of the number of convolutions that the layer would apply. The same filters are then used for all detection-cores without any fine-tuning.

In general, image-candidates are of several shapes. Building a model for each shape ends by creating and uploading a considerable number of detection-cores. We decided to build a limited number of detection-cores, one for each group of image-candidate that are similar in shape.

We assume that camera parameters, camera tilt angle, traffic density, and the number of lanes may change from one installation to another. We then consider the number of detection-cores as a tunable parameter for each installation. Still, generically, we study car shapes (as it is the smallest object to be detected) to conclude the dimensions of the biggest car. We set the height of the smallest detection-core (the detection-core with the smallest input shape) to this value and the width equal to the maximum width of a lane. The input shapes of other models are defined by combining all the possible multiples of widths or heights until reaching the shape that covers the entire frame. And then, all cases of heavy goods vehicles and vehicle densities are covered.

### 4.3. The Overall Architecture

**Figure 6:**
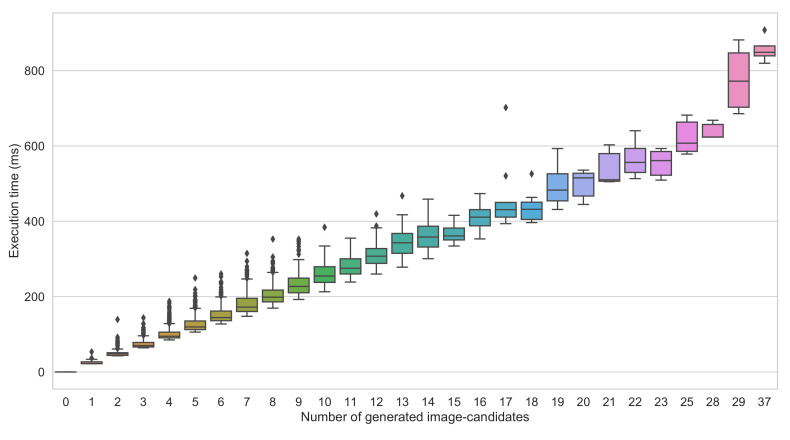
Illustration of the impact of the number of image-candidates on the execution time of the proposed framework (using YOLOv5l as base model of the detection-cores).

**Figure 7:**
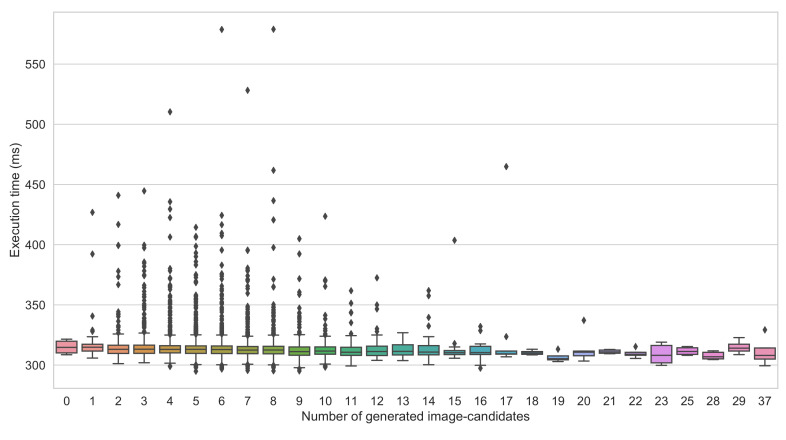
Illustration of the execution time distribution of the conventional method (YOLOv5l) versus the number of image candidates of the proposed framework.

The framework takes as input a video frame. It first applies the BS using the GMM algorithm. The GMM parameters were experimentally tuned (e.g., history size set to 100) to make it more sensitive to eliminate the false negatives (undetectable vehicles). The framework analyzes the obtained binary image using a blob finder looking for a group of white pixels (i.e., moving objects); in the experimental settings, the group of pixels is set to be at least 30 × 30. If no blob is detected, the frame is considered empty, and the algorithm processes a new video frame. Otherwise, the blobs are used to extract the associated parts from the original image; after shape-normalization, each image part (image-candidate) is fed to the appropriate detection-cores.The BS-based module may produce a considerable number of image-candidates, thus leading to high execution time. We found that the execution time of the proposed framework when the number of image-candidate is too high exceeds that of the conventional approach. As illustrated in Figure 6, the proposed framework’s execution time corresponding to 25 image-candidates is around 600 ms/image, whereas the conventional method’s execution time is around 310 ms/image regardless of the number of proposed image-candidates (see Figure 7). This motivates a judicious choice of the maximum number of image-candidates, denoted by T, for which the proposed framework remains useful. Hence, in the experimental setup of our study, we set T=10. When the number of the generated image-candidates exceeds *T*, the framework switches to the conventional approach by feeding the whole original image as a unique image-candidate. When not exceeding *T*, we compare the resulting image-candidates shapes to the prespecified detection-cores’ input shapes and select the suitable detection-core for every image-candidate. The suitable detection-core refers to the one with the smallest input shape that is greater than the considered image-candidate shape. As described before, we apply the shape-normalization technique to the image-candidate to get the input shape required by the selected detection-core. We run the detection-core on the image-candidate, which outputs the class and relative coordinates of all vehicles in the image-candidate space (with the (0,0) coordinates being at the upper left corner of the image-candidate); we then obtain their absolute coordinates in the original image space (with the (0,0) coordinate being at the upper left corner of the original image). The overall architecture of the proposed framework is illustrated in Figure 3.

## 5. Tests and Results

### 5.1. Dataset

To evaluate the performance of our framework under different conditions, we carried out experiments on a public dataset, namely the Highway Dataset [26]. It was collected using 23 surveillance cameras installed at the height of 12 m. Thus, the vehicles in the collected images have different scales. The dataset captured images from different scenes, on different times, and in different lighting conditions. The vehicles are classed into three categories: cars, buses, and trucks; The dataset contains 11,129 images.

### 5.2. Results

**Figure 8:**
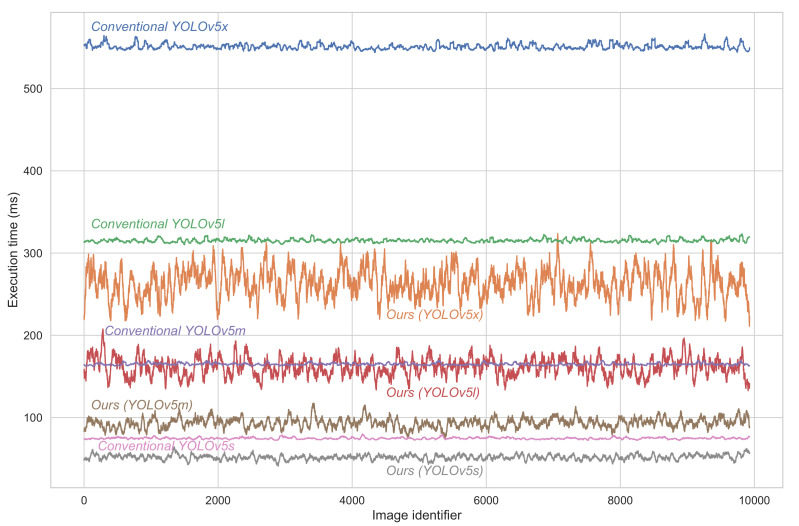
Comparison between the execution times of the proposed framework (ours) and the conventional detection algorithm using YOLOv5 architectures (YOLOv5s, YOLOv5m, YOLOv5l, and YOLOv5x).

In this study, we investigated the four architectures of YOLOv5, namely YOLOv5s (smallest), YOLOv5m, YOLOv5l and YOLOv5x (largest) [25], as base models of the detection-cores. The latter were pre-trained on the COCO dataset. The evaluation of the proposed framework measures the execution time and detection reliability. A true positive detection refers to the case where the vehicle type (i.e., class) is predicted correctly and the intersection over union (IoU) with the ground-truth bounding box is greater than 0.5 (AP@50 metric). If the object is misclassified, the result is referred to a false negative. Additionally, if the IoU is smaller than 0.5, we consider this as a false positive. In addition, we calculate the AP using different values of IoU (from 0.50 to 0.95 in 0.05 increments). The average of these APs is denoted by mAP (0.5:0.95). We performed the testing on an ASUS Laptop with a CPU of i7-5500U @ 2.40 GHz × 2, and a RAM of DDDR4 8 GB, using 64-bit Ubuntu 18.04 LTS. Figure 8 illustrates the execution time distribution of the proposed framework compared to the conventional method. The experiments were conducted on approximately 10,000 relevant images from the Highway Dataset [26]. As can be seen, the proposed framework significantly reduces the execution time for the four architectures. This reduction reached 30.6%, 43.6%, 48.7% and 52.2% when using YOLOv5s, YOLOv5m, YOLOv5l, YOLOv5x, respectively. A comparison of the proposed framework against the conventional method in terms of accuracy and execution time is presented in Table 2 on the Highway dataset. It can be seen that the proposed framework preserves the same accuracy as the conventional CNN-based detection method while reducing the execution time. Moreover, the deeper the network, the more important the margin. thus, the proposed framework is convenient for time-constrained monitoring applications that use CNN-based object detection.

## 6. Conclusions

This paper introduced a robust low-cost CNN-based moving vehicle detection framework combining Background Subtraction and Deep Learning. The proposed approach reduces the model complexity and running time. The proposed framework applies concise convolutions only over image regions containing vehicles. These regions were identified using the GMM algorithm. The proposed framework was tested on a large-scale public dataset. The framework preserved the same accuracy as the conventional CNN-based detection method while reducing the execution time by about 30.6% and 52.2% when using the YOLOv5s and YOLOv5x, respectively, as base models of the detection-cores.

## Figures and Tables

**Table 1:** Architecture Comparison of the detection-core 1 with the detection-core 2 having the same set of filters and different input shapes (few layers from YOLOv5s architecture).

Layer	Kernel Shape	Detection-Core 1	Detection-Core 2
Input	-	(96 × 64 × 3)	(640 × 384 × 3)
Conv. 2D	(3 × 3 × 32)	(48 × 32 × 32)	(320 × 192 × 32)
Conv. 2D	(3 × 3 × 64)	(24 × 16 × 64)	(160 × 96 × 64)
Conv. 2D	(1 × 1 × 32)	(24 × 16 × 32)	(160 × 96 × 32)
Conv. 2D	(1 × 1 × 32)	(24 × 16 × 32)	(160 × 96 × 32)
Conv. 2D	(3 × 3 × 32)	(24 × 16 × 32)	(160 × 96 × 32)
Conv. 2D	(1 × 1 × 32)	(24 × 16 × 32)	(160 × 96 × 32)
Conv. 2D	(1 × 1 × 64)	(24 × 16 × 64)	(160 × 96 × 64)
Conv. 2D	(3 × 3 × 128)	(12 × 8 × 128)	(80 × 48 × 128)

**Table 2:** Detection performance comparison using the AP@50 metric, the mAP (0.5:0.95) and the mean execution time per image (in millisecond) using four architectures.

Scenario	Params	Conventional			Ours		
		AP@50	mAP ${}_{\left(0.5:0.95\right)}$	Time	AP@50	mAP ${}_{\left(0.5:0.95\right)}$	Time
YOLOv5s	7.3 M	58.11	38.03	75	58.11	38.03	52
YOLOv5m	21.4 M	64.66	46.72	165	64.66	46.72	93
YOLOv5l	47.0 M	62.72	43.89	315	62.72	43.89	162
YOLOv5x	87.7 M	59.4	40.79	551	59.4	40.79	263

## Data Availability

The source code of this study is openly available at github.com/CharZakaria/Concise_Convs (accessed on 7 December 2021).
